# Supplementary material for: End Sequence Analysis Toolkit (ESAT) expands the extractable information from single-cell RNA-seq data
Source: Genome Res. 2016 Oct;26(10):1397–410. doi: 10.1101/gr.207902.116 (PMC5052061; doi:10.1101/gr.207902.116)
Supplement: Supplemental Material [file supp_gr.207902.116_Supp_Materials.tar.gz › ESAT-esat_v0.1/doc/html/namespacebroad_1_1pda_1_1rap_1_1misc.html]

ESAT: Package broad.pda.rap.misc


ESAT


- Main Page
- Related Pages
- Packages
- Classes
- Files

- Packages

All Classes Namespaces Files Functions Variables Enumerator Pages

- broad
- pda
- rap
- misc

Classes

Package broad.pda.rap.misc

|  |  |
| --- | --- |
| Classes | |
| class | FindEnrichedDepletedRegions |
|  | |


---

Generated on Fri Aug 15 2014 15:11:01 for ESAT by  

 1.8.7
